# Role of fuel's mixture on photocatalytic performance of g-C_3_N_4_/TiO_2_ nanocomposites

**DOI:** 10.1016/j.heliyon.2024.e40464

**Published:** 2024-11-16

**Authors:** S. Delafrouz, M. Hasheminiasari, S. Alamolhoda, S.M. Masoudpanah

**Affiliations:** School of Metallurgy & Materials Engineering, Iran University of Science and Technology (IUST), Tehran, Iran

**Keywords:** G-C_3_N_4_, TiO_2_, Photodegradation, Solution combustion, Photocatalysis

## Abstract

In this study, the main objective was to improve the photocatalytic performance of TiO₂ and g-C₃N₄ under visible light irradiation by the creation of g-C_3_N_4_/TiO_2_ heterojunction, where the solution combustion method was utilized to synthesize nanocomposites. The g-C_3_N_4_/TiO_2_ nanocomposites were fabricated with a 9:1 wt ratio of g-C₃N₄ to TiO₂ for investigation of the effects of different fuels, including urea, glycine, citric acid, and their mixtures, on the photocatalytic performance of the g-C₃N₄/TiO₂ nanocomposites. The nanocomposites were characterized using various techniques such as diffuse reflectance spectroscopy (DRS), photoluminescence (PL) spectroscopy, (BET) surface area analysis, field emission scanning electron microscopy (FESEM), Raman spectroscopy, and X-ray diffraction (XRD). The nanocomposite synthesized using a mixture of citric acid and urea (TCN-CA/U) depicted 97 % degradation of MB within 2 h of visible light exposure, demonstrating significantly enhanced photocatalytic efficiency. The degradation constant rate of TCN-CA/U (0.02971 min⁻^1^) was approximately 3 times higher than that of pure g-C₃N₄ (0.00952 min⁻^1^) and 13.5 times higher than that of TiO₂ (0.00218 min⁻^1^). Furthermore, the TCN-CA/U nanocomposite showed high degradation efficiencies for Rhodamine B (RhB) (91 %) and Methyl Orange (MO) (61 %) under the same conditions. The observed improvement in photocatalytic performance attributed to several factors, including a fourfold increase in specific surface area (52.5 m^2^/g) compared to g-C₃N₄ (12.2 m^2^/g), a significant reduction in electron-hole pair recombination, and a narrowed band gap energy of 2.57 eV, which played a crucial role in enhancing visible light absorption.

## Highlights

• Solution combustion was used to make g-C_3_N_4_/TiO_2_ nanocomposites with enhanced photocatalytic properties.
• Fuel type and mixture were studied to synthesize a g-C_3_N_4_/TiO_2_ composites with exceptional photocatalytic response.
• A mixture of urea and citric acid showed an order of 13 times improvement in photocatalytic activity.

## 1 Introduction

Pollutant levels in the environment have significantly increased since the start of industrialization. Many chemicals that pose serious threats to human health and ecosystems are released into our natural water sources, including pesticides, dyes, endocrine-disrupting compounds, medicines, and personal care items [[1], [2], [3]]. Recently, significant efforts have been made to introduce new solutions for eliminating organic contaminants [4]. These techniques include advanced oxidation processes (AOPs), membrane filtration, adsorption, biodegradation, and bio-sorption [5,6]. Among AOPs, photocatalysts have demonstrated a high degree of effectiveness in transforming pollutants into safe, eco-friendly, and comparatively non-toxic chemicals without producing any secondary pollution [5,[7], [8], [9], [10]]. The capacity of the photocatalyst to absorb photons with energies equal to or greater than the band gap energy determines the effectiveness of photocatalyst. Electron-hole pairs are created, move to the surface of the photocatalyst, and react with water (H_2_O), oxygen (O_2_), and hydroxyl groups to produce reactive oxygen species (ROS) that have strong oxidation capabilities, such as hydroxyl radicals (·OH) and superoxide radical anions (·O^2−^). The principal species accountable for the breakdown of persistent organic contaminants in wastewater is the ROS [9]. Metal sulfides or oxides like TiO_2_, MoS_2_, ZnO, ZnS, WO_3_, V_2_O_5_, and Bi_2_WO_6_ are examples of semiconductor photocatalysts. Metal-free semiconductors like g-C_3_N_4_ are also utilized in the photocatalysis process [11].

Titanium dioxide (TiO_2_) is now the material of choice for photocatalysis because of its remarkable properties, which include chemical stability, non-toxicity, and affordability [[12], [13], [14],^15^]. It is a great option for a number of photocatalytic uses, including the creation of hydrogen via water splitting, the purification of water and air, the development of self-cleaning surfaces, and the advancement of solar cell technology [16,17]. However, TiO_2_'s wide band gap energy (>3.0 eV) limits its photocatalytic potential to the ultraviolet (UV) region. The majority of artificial light sources and sunlight emit visible light, which is noteworthy because less than 5 % of visible light is ultraviolet (UV) [18,19,20]. To address this problem, researchers have tried to improve TiO_2_ performance by creating heterojunctions like CdS, WO_3_, and g-C_3_N_4_. To increase photocatalytic efficiency, this technique seeks to decrease the rate of electron-hole pair recombination and increase photon absorption [[21], [22], [23]].

Graphitic carbon nitride (g-C_3_N_4_) has garnered significant interest as a photocatalyst that responds to visible light. Aside from being non-toxic, this two-dimensional, metal-free material is also easily synthesized, inexpensive, has a narrow band gap (Eg = 2.7 eV), and is environmentally friendly [[24], [25], [26], [27],^28^]. The fact that the maximum occupied molecular orbital (HOMO) of g-C_3_N_4_ is less than the conduction band (CB) of typical semiconductor photocatalysts like TiO_2_ and ZnO (1.12 eV) is one of its most alluring characteristics [29,30]. Nitrogen-rich molecular precursors such as urea, cyanamide, dicyandiamide, melamine, or thiourea are thermally polycondensed to produce g-C_3_N_4_ [31]. Due to these characteristics, g-C_3_N_4_ is widely used in visible light chemical synthesis, water splitting, CO_2_ reduction, and organic pollutant degradation [32]. Nevertheless, bulk g-C_3_N_4_ materials have a high rate of photogenerated carrier recombination and a low specific surface area—typically less than 20 m^2^ g^−1^—when used for photocatalysis [32,33].

Considering the matched band positions of g-C_3_N_4_ and TiO_2_, as well as the distinctive layered structure of g-C3N4 that offers a superb platform for TiO_2_ attachment, a TiO_2_/g-C_3_N_4_ heterostructure can be created through both type II and Z-scheme mechanisms [34]. Constructing the TiO_2_/g-C_3_N_4_ heterostructure can be resulted in a decrement in the recombination rate of photoinduced electron-hole pairs and the band gap energy reduction, thereby it may cause extention to the light absorption region [35,36,37].

Metal oxide synthesis can now be accomplished more economically and effectively with the introduction of solution combustion synthesis (SCS) [38,39]. This process is based on the exothermic reaction that releases thermal energy and produces gaseous byproducts when oxidizing agents like nitrates, acetates, and chlorides combine with organic fuels like urea, glycine, and citric acid [40]. Notably, the type and content of the fuel mostly determine the powder's properties, such as phase, morphology, particle size, and specific surface area [41]. The process of burning organic fuels releases heat necessary for the creation of products and helps break down larger particles into porous and spongy powders [42]. However, blending multiple fuels can yield more efficiency than using just one fuel, providing efficient control over the temperature at which adiabatic combustion occurs as well as the kind and volume of gaseous products that are released [40]. On the other hand, the SCS technique suffers from primary limitations, including issues with powder agglomeration, potential difficulties in controlling powder morphologies, and the presence of residual carbonaceous and organic impurities due to incomplete combustion [43].

In this study, the in-situ fabrication of TiO_2_ on g-C_3_N_4_ was achieved via the solution combustion method. This technique releases a large volume of gases during combustion, which enhances the specific surface area of the resulting nanocomposite. Additionally, the significant energy released during the process influences the particle size of TiO_2_. To explore the effects of different fuels on the photodegradation efficiency of the g-C_3_N_4_/TiO_2_ heterojunction for methylene blue (MB) under visible light, three fuels—glycine, urea, and citric acid—were employed. For further improvement in photocatalytic performance, combinations of fuels such as citric acid-urea and citric acid-glycine were also utilized. The morphological properties and photocatalytic activity of the nanocomposite were studied, revealing that photocatalytic efficiency varied based on the type of fuel used.

## 2 Experimental procedures

Tetrabutyl titanate (C_16_H_36_O_4_Ti, purity ≥98 %), ammonium nitrate (NH_4_NO_3_, purity ≥95 %), urea (CH_4_N_2_O, purity ≥99 %), glycine (C_2_H_5_NO_2_, purity ≥99 %), citric acid (C_6_H_8_O_7_, purity ≥99 %), and melamine (C_3_H_6_N_6_, purity ≥99 %) were the substances employed in our experiments without any additional purification. These were all acquired from the Merck Corporation.

## 3 Synthesis of photocatalysts

To create g-C_3_N_4_, a muffle furnace was used to heat 25 g of melamine powder from room temperature to 550 °C at a rate of 10 °C per minute. Before allowing it to cool within the furnace, it was kept at the specified temperature for 2 h [44].

The solution combustion approach was utilized to create the g-C_3_N_4_/TiO_2_ nanocomposites. To accomplish a 1:9 wt ratio of TiO_2_ to g-C_3_N_4_, 1.35 g of graphitic carbon nitride was added to 40 mL of distilled water. Tetrabutyl titanate served as the TiO_2_ precursor, ammonium nitrate served as the oxidant, and several fuels, including urea, glycine, and citric acid, were dissolved into the solution and subjected to a 15-min ultrasonic bath. The mixture was then agitated and allowed to evaporate at 80 °C until it was entirely dry. The dry gel was heated on a hotplate to start the combustion reaction. After that, the powder was allowed to cool to ambient temperature inside a muffle furnace, where it was calcined for an hour at 450 °C.

The labels on the nanocomposites were "CA" for citric acid, "U" for urea, and "Gly" for glycine, according to the kind of fuel that was utilized. For the sake of simplicity, the g-C_3_N_4_/TiO_2_ nanocomposite was labeled "TCN".

For simplicity, the g-C_3_N_4_/TiO_2_ nanocomposite was generally labeled as 'TCN,' with specific labels following a hyphen to indicate the fuel used: TCN-U for Urea, TCN-CA for Citric acid, TCN-Gly for Glycine, TCN-CA/Gly for the combination of Citric acid and Glycine, and TCN-CA/U for the combination of Citric acid and Urea.

## 4 Characterization methods

Using a variety of methods, including X-ray diffraction (XRD) and Cu Kα radiation with a wavelength of 1.5418 Å, patterns were created on the synthesized samples. The Bruker D8 ADVANCE apparatus was used to construct the patterns. In addition, the Scherrer equation is a useful tool for analyzing the size of titanium oxide crystallites on graphitic nitride carbon. Measurements of Raman spectroscopy were performed using an Nd: YAG laser source on an XploRA PLUS spectrophotometer (Horiba, Japan). With a MIRA3 scanning electron microscope (TESCAN, Czech Republic), we were able to obtain information on particle size and elemental distribution. The pore size distribution, pore volume, and specific surface area were measured using the BELSORP Mini IIx equipment (MICROTRAC MRB, Japan). Band gap energy values were obtained through the use of a UV–visible 52550 spectrophotometer (Shimadzu, Japan) to record diffuse reflectance spectra. PL spectra were obtained at a wavelength of 325 nm using a Cary Eclipse fluorescence spectrophotometer (Agilent, USA) to examine charge separation.

## 5 Photocatalytic characterization

0.1 g samples were exposed to 5 ppm solutions of methyl orange (MO), rhodamine B (RhB), and methylene blue (MB) in order to assess the photocatalytic activity against dyes. The photocatalytic degradation was carried out with two 100 W Xenon lamps to provide visible light illumination and a 420 nm cutoff filter to block UV radiation. An hour of darkness was applied to the solutions prior to light exposure in order to bring the dyes' adsorption/desorption equilibrium. Based on the following formulas, the degradation rate and apparent rate constant (k) were calculated:

(1) $$Degradation\;rate=\left[\left(C_{0}-C\right)/C_{0}\right]\times 100\%$$

(2) $$\ln\left(C/C_{0}\right)=-kt$$

For the MB, RhB, and MO aqueous solution, C_0_ and C stand for the concentration at adsorption-desorption equilibrium and the concentration following a particular exposure period. The exposure time to visible light is represented by the rate constants k and t.

Prior to perform photocatalytic tests on the TCN-CA/U nanocomposite, 1 mM EDTA-2NA, p-BQ, and IPA were added to the reaction solution in order to examine the visible light-induced photocatalytic mechanism of TCN nanocomposites.

## 6 Results and discussion

A significant quantity of CO_2_, N_2_, and H_2_ were released as byproducts during the combustion process, and the amounts of these chemicals were calculated using a chemical reaction inspired by propellant chemistry:

(3) 104NH_4_NO_3_+ 2C_16_H_36_O_4_Ti+ 2C_6_H_8_O_7_**→**2TiO_2_+44CO_2_+252H_2_O+104N_2_

(4) 105NH_4_NO_3_+2C_16_H_36_O_4_Ti+2C_2_H_5_NO_2_**→**2TiO_2_+36CO_2_+251H_2_O+106N_2_

(5) 102NH_4_NO_3_+2C_16_H_36_O_4_Ti+2CH_4_N_2_O**→**2TiO_2_+34CO_2_+244H_2_O+103N_2_

(6) 123NH_4_NO_3_+2C_16_H_36_O_4_Ti+ 2C_6_H_8_O_7_+2C_2_H_5_NO_2_**→**2TiO_2_+48CO_2_+295H_2_O+124N_2_

(7) 120NH_4_NO_3_+2C_16_H_36_O_4_Ti+2C_6_H_8_O_7_+2CH_4_N_2_O**→**2TiO_2_+46CO_2_+288H_2_O+121N_2_

**Fig. 1:**
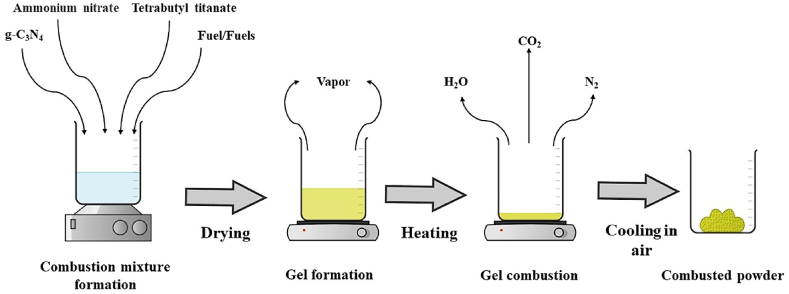
TCN solution combustion schematic route.

**Fig. 2:**
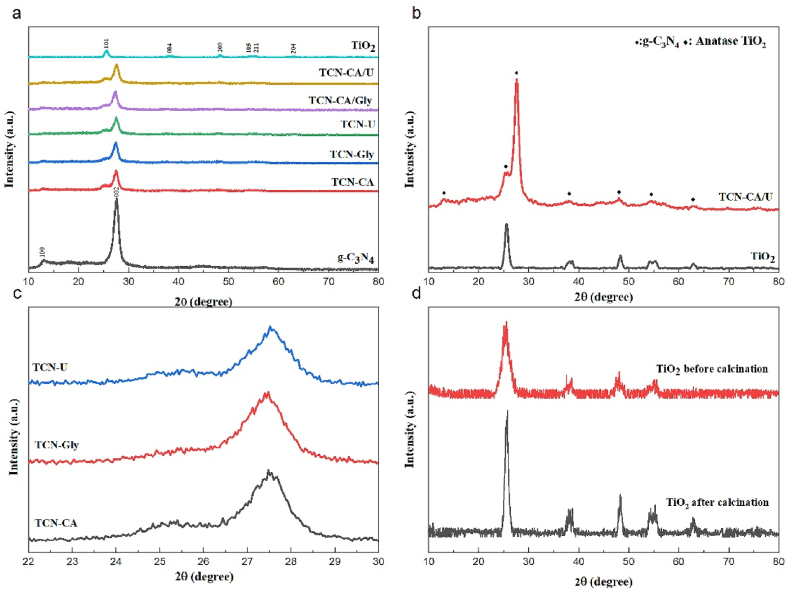
(a) XRD patterns of the synthesized samples (b)XRD patterns of TiO2 AND TCN-CA/U (c) XRD pattern of TCN-CA, TCN-U, and TCN-Gly for comparison intensity of (101) plane (d) XRD patterns of TiO_2_ before and after calcination.

The structural properties of pristine g-C_3_N_4_, TiO_2_, and nanocomposite materials are shown by the XRD patterns in Fig. 2a, (see. Fig. 1) Specifically, the XRD pattern of g-C_3_N_4_ reveals two strong reflections at 2θ = 13.1° and 27.7°, which indicate that the conjugated aromatic units are stacked in the interlayers (002) plane and the structural units are stacked within the same plane (100) [45,46]. (JCPDS 87–1526).

In contrast, six separate diffraction peaks can be seen in the XRD pattern of pure TiO_2_ at about 25.0°, 37.9°, 48.2°, 54.4°, 55.3°, and 62.8°. These peaks correspond to the crystalline planes of anatase TiO_2_ (JCPDS 01-073-1764) at positions (101), (004), (200), (105), (211), and (204), respectively.

The titanium dioxide anatase phase coexists with graphitic carbon nitride in the produced nanocomposite samples, as determined by XRD pattern analysis. The nanocomposite samples display a comparatively great intensity in the graphitic carbon nitride peaks, as demonstrated in Fig. 2b. The minute crystallinity of TiO_2_ nanoparticles and the use of a 9:1 wt ratio of graphitic carbon nitride to titanium oxide during the manufacturing process are responsible for this increased intensity.

The materials' crystallite sizes were ascertained using the Scherrer equation. The broadening of a peak in a diffraction pattern and the size of crystallites in a solid are related, according to this formula:

(8) d=0.9λcos(θ)/β

In this formula, d stands for crystalline size, λ for X-ray radiation wavelength, β for full-width half maximum value (FWHM) in radians, and θ for diffraction angle [47]. Table 2 presents the crystallite sizes of TCN nanocomposites and pure TiO_2_, calculated by applying Scherrer's equation to the dominant (101) plane of anatase TiO_2_.

**Table 1:** The measurement of specific surface area, volume, and porosity size of g-C_3_N_4_, TiO_2_, and TCN-CA/U nanocomposite samples.

Sample	Specific surface area (m^2^.g^−1^)	Pore volume (cm^3^.g^−1^)	Average pore diameter (nm)
g-C_3_N_4_	12.955	0.0771	23.828
TiO_2_	19.170	0.0658	24.312
TCN-CA/U	52.505	0.1716	13.075

**Table 2:** The measurement of band gap energy and crystallite for g-C_3_N_4_, TiO_2_, and TCN nanocomposites.

Sample	Band gap energy(eV)	Crystallite size(nm)
TiO_2_ (before calcination)	–	5.79
TiO_2_ (after calcination	3.06	10.15
TCN-CA	2.64	7.42
TCN-GLY	2.6	6.83
TCN-U	2.71	6.98
TCN-CA/U	2.57	7.39
TCN-CA/GLY	2.53	7.21
g-C_3_N_4_	2.67	–

Differences in the intensity of the titanium oxide anatase peaks have been detected, as Fig. 2c demonstrates, and these differences are related to the kind of fuel that was utilized in the synthesis process. The discrepancies in intensity point to variations in the crystallinity of the titanium oxide particles that were synthesized by solution combustion. Interestingly, the sample with the largest crystallite size was produced by citric acid as a fuel source.

Additionally, because of the increased structural order, the crystallinity of the titanium dioxide particles improves as the calcination temperature rises. The variations in TiO_2_ before and after calcination at 450 °C are displayed in Fig. 2d. Sharper and smaller X-ray peaks are the outcome of this improvement. Higher calcination temperatures result in larger crystallites, which suggest a notable improvement in TiO_2_ crystallinity. Grain boundary flaws seem to be removed during the high-temperature calcination process, which further enhances the crystallinity of TiO_2_ nanoparticles.

Moreover, these results demonstrate how important the calcination procedure and the fuel type are in raising the crystallinity of TiO_2_. In order to increase the size of crystallites and overall crystallinity, calcination is necessary. This increases the suitability of TiO_2_ powder for a variety of applications. This is especially helpful for high-performance applications that need high crystallinity, such as photocatalysis.

**Fig. 3:**
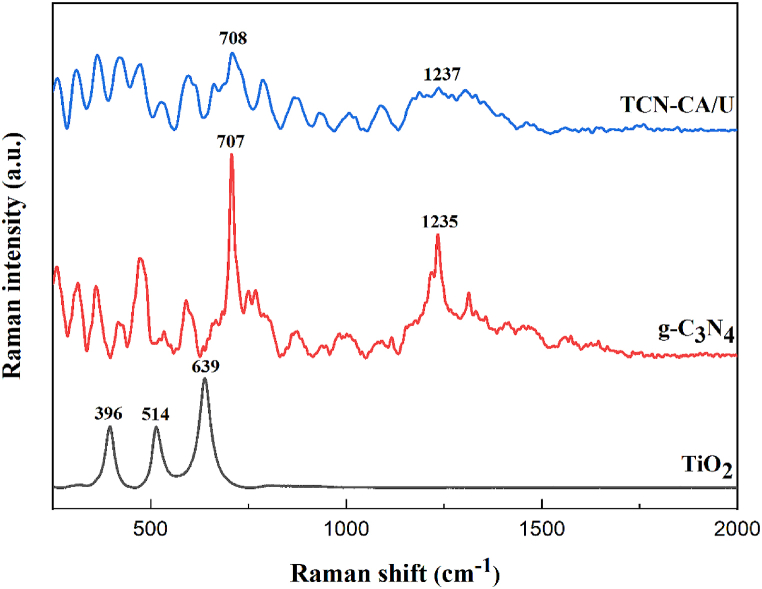
Raman spectra of TiO_2_, g-C_3_N_4_ and TCN-CA/U nanocomposite.

Raman spectroscopy is necessary to investigate whether phases may exist in the samples. By contrasting the measured vibration modes with the body of known literature, the makeup of each phase was determined. Fig. 3 shows that the TiO_2_ anatase phase is characterized by three distinct peaks at 396, 515, and 639 cm^−1^. In Raman spectroscopy, these peaks are linked to Ti-O vibrational modes observed for the anatase phase [48,49]. Moreover, several bands in the 700–1500 cm^−1^ region are visible in the Raman spectra of g-C_3_N_4_, suggesting the presence of graphitic carbon nitride [50]. Notably, the ring breathing modes of s-triazine are represented by the peak at 707 cm^−1^, whereas the g-C_3_N_4_ crystal's lattice vibration is represented by the peak at 1235 cm^−1^. Wide vibrational bands are visible in the Raman spectra of the produced TCN-CA/U nanocomposite, as shown in Fig. 3. The reduction in the intensity of the characteristic peaks of TiO_2_ and g-C_3_N_4_ [51] indicates a correlation between the quantity of these vibrations and the ratio of TiO_2_ to g-C3N_4_. New chemical bonds at the g-C_3_N_4_-TiO_2_ interface cause alterations in the Raman spectra (200-500 cm^−1^) for the produced TCN-CA/U nanocomposite. However, because of the 9:1 wt ratio of g-C_3_N_4_ to anatase phase for produced nanocomposites, response signals of g-C_3_N_4_ are so strong that the anatase phase is hardly noticeable. Significantly, a red shift showed interactions between g-C_3_N_4_ and TiO_2_, emphasizing the accomplishments of the TCN nanocomposite synthesis [52].

**Fig. 4:**
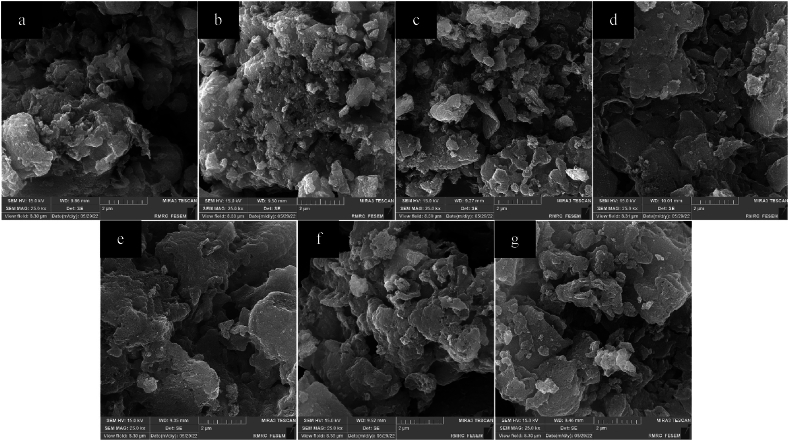
SEM images of (a)g-C_3_N_4_ (b)TiO_2_ (c)TCN-CA (d)TCN-U (e)TCN-Gly (f)TCN-CA/Gly and (g)TCN-CA/U.

The FESEM images of generated TCN nanocomposites, pristine TiO_2_, and bulk g-C_3_N_4_ are displayed in Fig. 4. Fig. 4a and b, respectively, depict the bulk sheet-like structure of g-C_3_N_4_ and the agglomerated spherical-like particles of pure TiO_2_. This finding is consistent with findings from previous research [53,54].

Fig. 4(c–g) illustrate the non-uniform distribution of spherical-like TiO_2_ particles on the g-C_3_N_4_ surface in the nanocomposites sample. This dispersion pattern is significant because it suggests that g-C_3_N_4_ contributes to the partial inhibition of TiO_2_ nanoparticle agglomeration. This dispersion enhances the physical stability of the nanocomposite and facilitates the interfacial contact between the TiO_2_ and g-C_3_N_4_ particles. By effectively separating electron-hole pairs during photocatalysis, these interactions greatly improve the photocatalytic efficiency of the nanocomposite.

These samples also show different amounts of porosity, which is a property that depends on the kind of fuel utilized in the synthesis process. Increased porosity is linked to shorter combustion times that result in bigger particle formation and a more noticeable emission of gaseous pollutants during combustion. The sample that was created by using citric acid as fuel is particularly porous. This characteristic is the result of combustion's increased production of gas byproducts. On the other hand, samples synthesized using urea and glycine fuels have lower porosity values. Specifically, when two fuels are burned together, more gasses are released during the combustion process, which creates a more porous structure than when one fuel is burned alone.

Varying fuel properties lead to different breakdown temperatures, which cause variations in TiO_2_ particle sizes. Larger TiO_2_ particles are formed when citric acid is used as a fuel with a lower decomposition temperature. In comparison, glycine produced smaller particles due to its greater breakdown temperature. Because of the way the two fuels interact to affect the decomposition temperatures, using mixed fuels introduces additional complexity [55]. Interestingly, the TiO_2_ particles obtained from TCN-CA/Gly are smaller than those obtained from TCN-CA/U. This emphasizes how crucial the temperature of decomposition is in dictating the sizes of the particles during the synthesis process. Notably, these outcomes coincide with the information obtained by the Scherer equation.

Moreover, as was already indicated, samples created by combining fuel also showed the presence of bigger particles when citric acid was added. Porosity and the non-uniform distribution of TiO_2_ on g-C_3_N_4_ layers were both clearly visible. In conclusion, the porosity, agglomeration, and structural characteristics of TCN nanocomposite materials are strongly influenced by the fuel selection made during the solution combustion synthesis process. The specific features of the fuel employed are intricately linked to the dispersion of TiO_2_ particles on the g-C_3_N_4_ surface, the level of porosity, particle size, and the combustion circumstances.

**Fig. 5:**
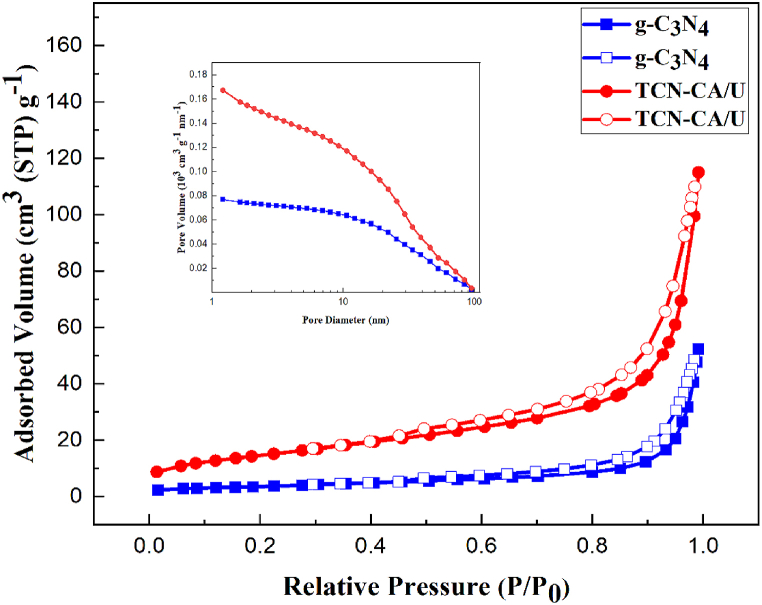
Adsorption-desorption curve and pore size distribution of g-C_3_N_4_ and TCN-CA/U.

The nitrogen adsorption-desorption isotherms for TCN-CA/U and g-C_3_N_4_ are shown in Fig. 5. It's significant to remember that both materials display type IV isotherms in accordance with IUPAC categorization, indicating that mesopores usually have sizes between 2 and 50 nm [56]. In these isotherms, the hysteresis loops that belong to the H3 group are very fascinating. According to these loops, the pores in these materials can have a variety of morphologies, including parallel, slit-like, and open-ended tubular structures [57]. Moreover, the isotherms demonstrate significant adsorption at relative pressures (P/P_0_) close to 0.1, suggesting the existence of macropores as well as mesopores.

The pore size distribution, which was calculated on the adsorption branch of the isotherm using the Barrett-Joyner-Halenda (BJH) technique, is also shown in Fig. 5. The mesoporous nature of g-C_3_N_4_ and the TCN-CA/U nanocomposite is confirmed by the pore size distribution. The average BJH pore size of the TCN-CA/U nanocomposite is 10 nm, whereas the average pore size of pure g-C_3_N_4_ is less at 5.2 nm.

Specific surface area, pore volume, and average pore diameter are among the computed values from the BET study that are shown in Table 1. Notably, the TCN-CA/U nanocomposite (52.5 m^2^ g^−1^) exhibits a notable increase in specific surface area. This number is almost four times more than the pure g-C_3_N_4_ material's specific surface area (12.9 m^2^ g^−1^). In addition, the average diameter decreased, and the nanocomposite's pore volume doubled compared to g-C_3_N_4_. This accomplishment improves photocatalytic performance. The quick release of gases during solution combustion is responsible for these amazing changes in pore characteristics.

**Fig. 6:**
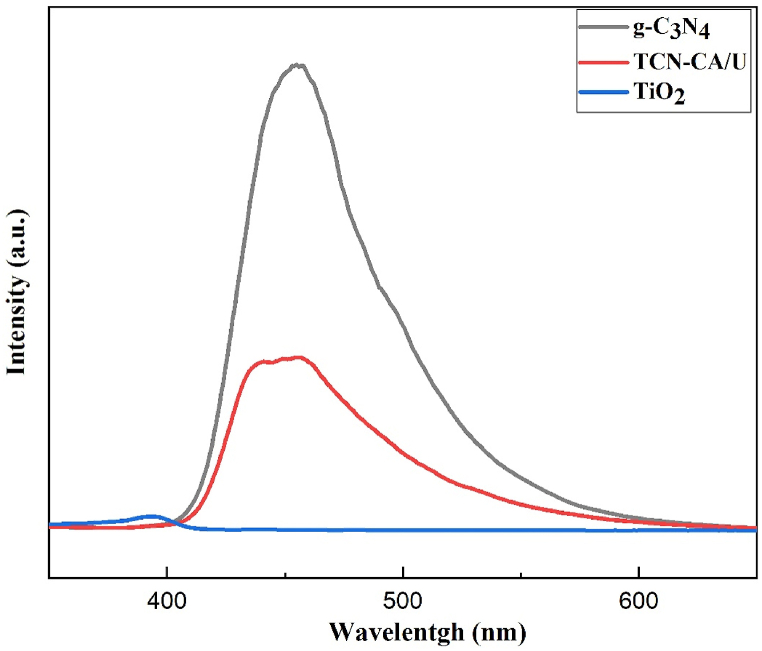
Photoluminescence spectra of TiO_2_, g-C_3_N_4_ and TCN-CA/U nanocomposite.

The photoluminescence spectra of three different materials—pure TiO_2_, g-C_3_N_4_, and the TCN-CA/U nanocomposite—are shown in Fig. 6. A wavelength of 325 nm was used to ignite each of these materials. At about 455 nm, which corresponds to the pure g-C_3_N_4_ band gap, the principal emission peak of graphitic carbon nitride is seen within this spectrum. On the other hand, the band gap of anatase TiO_2_ is aligned with the previous emission peak at around 390 nm.

The TCN-CA/U nanocomposite exhibits significantly lower emission intensity than pure g-C_3_N_4_ when these emissions are compared. A slower rate of electron-hole pair recombination within the nanocomposite is shown by this decrease in intensity. More efficient recombination suppression is shown by this drop in photoluminescence intensity, which in turn increases the photocatalytic activity of the nanocomposite in the visible light spectrum. This result is especially noteworthy for photocatalytic applications since obtaining better performance under visible light irradiation depends critically on effective charge separation. Interestingly, synthetic TiO_2_ produced via solution combustion had the lowest intensity, indicating that it was a highly effective photocatalyst that was only active in the UV region.

**Fig. 7:**
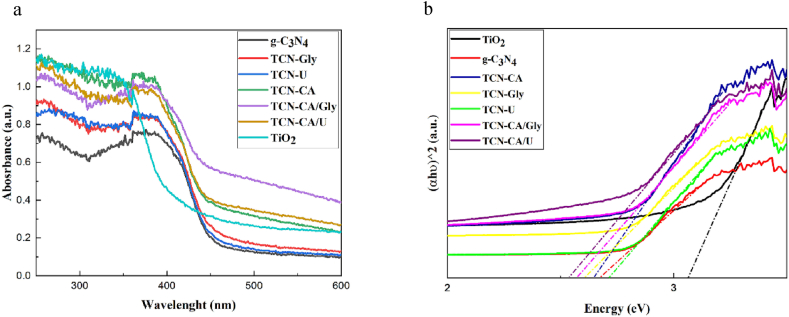
(a) UV–vis absorption spectra of g-C_3_N_4_, TiO_2,_ and TCN nanocomposites (b) transformed diffuse reflectance spectra of g-C_3_N_4_, TiO_2,_ and TCN nanocomposites.

Prior to evaluating the photocatalytic performance of g-C_3_N_4_, TiO_2_, and TCN nanocomposites, an investigation of their optical characteristics was conducted. One important signal that is directly related to the band gap energy of the semiconductor is the UV–vis absorption edge. The reflectance spectrum is shown in the corresponding Fig. 7a. Graphitic carbon nitride is notable for having an absorption edge at 456 nm. Furthermore, a prominent absorption peak corresponding to the absorption spectrum of TiO_2_ is detected in the UV (200–400 nm) range. This indicates that TiO_2_ responds relatively little in the visible light spectrum and functions as a semiconductor in the ultraviolet range. Remarkably, all produced nanocomposites have a common absorption edge of about 450 nm, which places them in the visible light area where they are effective photocatalysts.

A Tauc plot was used to measure the optical band gap energy of these samples. Equation [58] is used to calculate the band gap energy value.

(9) $${\left(\alpha hv\right)}^{n}=A\left(hv-Eg\right)$$

Where n takes on different values depending on the type of transition (n = 2 for direct allowed transitions, 2/3 for direct forbidden transitions, and 1/2 for indirect allowed transitions) and α stands for the absorption coefficient, hν for photon energy, A for material-specific constant, and Eg for the band gap energy [59]. The band gap values were computed by extrapolating the (αhv)^2^ against photon energy plots for direct transitions, as shown in Fig. 7b.

Based on the data shown in Table 2, the band gap energy of bulk graphitic carbon nitride is 2.67 eV. In contrast, titanium dioxide exhibits greater absorption with a bandgap energy of 3.06 eV than previously reported samples of anatase-phase titanium dioxide (3.2 eV) [58]. Depending on the particular fuel used, the nanocomposites made with different fuels, such as urea (2.71 eV), glycine (2.6 eV), and citric acid (2.64 eV), show band gap energy similar to graphitic carbon nitride. In the case of mixed fuels, the narrowing of the band gap observed in TCN-CA/Gly (2.53 eV) and TCN-CA/U (2.57 eV) is a result of improved light absorption. This effect can be attributed to the carbon dopants introduced during the combustion process, which may have created a synergistic effect, leading to more pronounced electronic states within the photocatalyst's band gap [54]. According to the molecular formulas of each fuel, Urea has the least carbon content, while Glycine has the most carbon. This higher carbon content in Glycine led to a narrower band gap in its presence, while the presence of Urea as fuel resulted in a band gap close to that of bulk graphitic carbon nitride.

Surprisingly, the fuel selection affects these energy levels, with urea and citric acid producing higher band gap energies than glycine. The light absorption zone is expanded by a fuel combination, most likely as a result of a more cooperative combustion process.

## 7 Photocatalytic performance

**Fig. 8:**
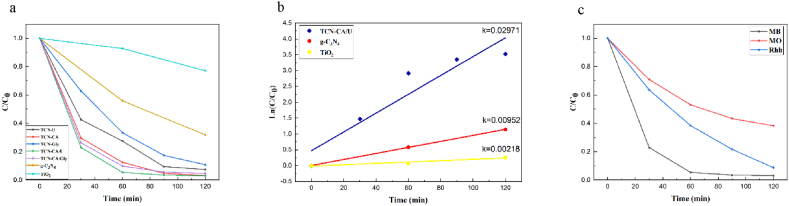
(a) photocatalytic performance of TiO_2_, g-C_3_N_4_, and TCN nanocomposites for the MB degradation under visible light (b) corresponding kinetic curves for TiO_2_, g-C_3_N_4,_ and TCN-CA/U nanocomposite (c) photocatalytic performances of TCN-CA/U nanocomposite for MB, RhB, and MO dyes.

The photocatalytic activity of TiO_2_, g-C_3_N_4_, and TCN nanocomposites was evaluated for the degradation of methylene blue (MB) under visible light irradiation at pH 6. The MB concentration drops with time when these photocatalysts are present, as seen in Fig. 8a. TiO_2_ only showed a 22.97 % degradation of MB after 120 min of exposure to visible light because of its band gap energy (>3 eV), which disqualifies it as a UV photocatalyst.

On the other hand, g-C_3_N_4_—which has a band gap energy of 2.67 eV—is widely known for its photocatalytic capabilities for visible light, demonstrating a degradation rate of 68.09 % during an equivalent time frame. Furthermore, the photocatalytic degradation of MB was considerably accelerated by the produced TCN nanocomposites. As compared to pure TiO_2_ and g-C_3_N_4_, all of the generated samples performed better in terms of MB degradation, highlighting the usefulness of the synthesized heterojunctions. TCN-CA/U outperformed the other nanocomposites in terms of degradation rate; photo degrading 97.03 % of MB in just 120 min out of those produced using various fuels.

The choice of fuel has a major impact on the finished product's properties. Chelating chemicals maintain a constant composition, encourage strong coordinate connections, and stop metal ions from precipitating. Because of its unique chelating properties and capacity to produce stable metal ions, citric acid performs better in this study's photocatalytic performance for TCN heterojunction when compared to glycine and urea when using a solution combustion approach. The three carboxyl groups in citric acid allow for regulated deposition, which increases the size of TiO_2_ particles on the g-C_3_N_4_ surface, promoting effective charge separation and improving photocatalytic activity [55]. Lower photocatalytic activity is the result of urea and glycine's inability to precisely control TiO_2_ deposition, unlike citric acid, despite their effectiveness as chelating agents. Due to the beneficial usage of two fuels, which results in increased gas release and a more synergistic combustion reaction, the photodegradation of MB in mixed fuel synthesis is improved in less than 1 h of visible light irradiation. This improves the efficiency of the degradation process.

The apparent rate constants and photocatalytic degradation values for the synthetic materials are shown in Table 3. Plotting Ln(C_0_/C) against visible light irradiation time for g-C_3_N_4_, TiO_2_, and TCN-CA/U is shown in Fig. 8b. Remarkably, as compared to bulk g-C_3_N_4_ and TiO_2_, the solution combustion approach produced a TCN-CA/U nanocomposite with higher reaction rates. After 120 min of exposure to visible light, the apparent rate constant for the most favorable sample, TCN-CA/U, showed an astounding 13.5-fold increase over pure titanium oxide, as shown in Fig. 8b. It also demonstrated a three-fold enhancement over graphitic carbon nitride in bulk.

**Table 3:** The measurement of MB degradation of g-C_3_N_4_, TiO_2_, and TCN nanocomposites under visible light irradiation.

Sample	Apparent constant	Degradation (%)	Time (min)
TiO_2_	0.00218	22.97	120
g-C_3_N_4_	0.00952	68.09	120
TCN-CA	0.02909	96.81	120
TCN-GLY	0.01913	89.22	120
TCN-U	0.02232	92.52	120
TCN-CA/Gly	0.02558	95.36	120
TCN-CA/U	0.02971	97.03	120

The degradation of several dyes at a 5 ppm concentration over time in response to the 0.1 g produced TCN-CA/U sample is shown in Fig. 8c. Three dyes—methylene blue (MB), methyl orange (MO), and rhodamine B (RhB)—were exposed to visible light for more than 120 min. The significant deterioration of methylene blue (MB) to an astonishing 97.03 % under these conditions indicates the extreme vulnerability of MB to degradation. However, Rhodamine B (RhB) degraded 91.24 % over the same time period, highlighting differences in the degradation mechanisms linked to these dyes' unique chemical structures. Methyl orange (MO), on the other hand, showed a lesser level of deterioration, at about 61 %.

The dyes' different levels of light sensitivity can be related to their different photodegradation efficiency. According to experimental results, dyes may be light-sensitive, which would lead to the excited electrons in the dyes moving into the conduction band (CB) of the photocatalyst. These excited electrons then react with oxygen to produce superoxide radicals, which are essential for breaking down the dye molecules. Among these dyes, there were notable differences in the values of the highest occupied molecular orbital (HOMO) energy gap, which were −10.128 eV for Rhodamine B, −10.494 eV for methylene blue, and −5.624 eV for methyl orange. The reason for RhB and MB's higher degradation rates can be attributed to their more favorable electron transport to the photocatalyst [60].

Table 4 presents a comparison of the photocatalytic efficiency of TiO_2_/g-C_3_N_4_ across different dyes, as reported in various studies, alongside the best result obtained in the present research. The data indicates that the photodegradation efficiency of dyes is enhanced when a heterojunction of TiO_2_/g-C_3_N_4_ is formed. This suggests that the formation of such a heterojunction is a viable, straightforward, and cost-effective method for improving photocatalytic efficiency.

**Table 4:** Comparison of photocatalytic performance of TiO_2_/g-C_3_N_4_ with the literature.

Catalyst amount (g)	Light source	Dye	Concentration (ppm) and volume (ml) of dye	Illumination time (min)	Degradation (%)	Ref.
0.1	Xenon lamp (350 W)	MB	10,20	550	≈90	[12]
0.04	Xenon lamp (350 W)	RhB	10,30	100	≈100	[21]
0.2	Xenon lamp (300 W)	RhB	10,50	240	63	[23]
0.1	Xenon lamp (300 W)	MB	10,100	180	79.7	[30]
0.05	Xenon lamp (300 W)	RhB	20,100	120	≈100	[61]
0.05	Xenon lamp (300 W)	MB	10,100	120	95	[62]
0.05	Xenon lamp (300 W)	RhB	10,100	120	96	[62]
0.1	Xenon lamp (200 W)	MB	5100	120	97.03	Present work
0.1	Xenon lamp (200 W)	RhB	5100	120	91.24	Present work

### 7.1 Possible photodegradation mechanism

To investigate the degradation mechanism during the photocatalytic process, scavenger experiments were conducted to identify the active species that play a crucial role. Isopropanol (IPA), para-benzoquinone (p-BQ), and ethylenediaminetetraacetic acid disodium salt dihydrate (EDTA-Na2) were used to scavenge hydroxyl radicals (O· H), superoxide radicals (O·2‐), and holes ($h^{+}$), respectively [63].

**Fig. 9:**
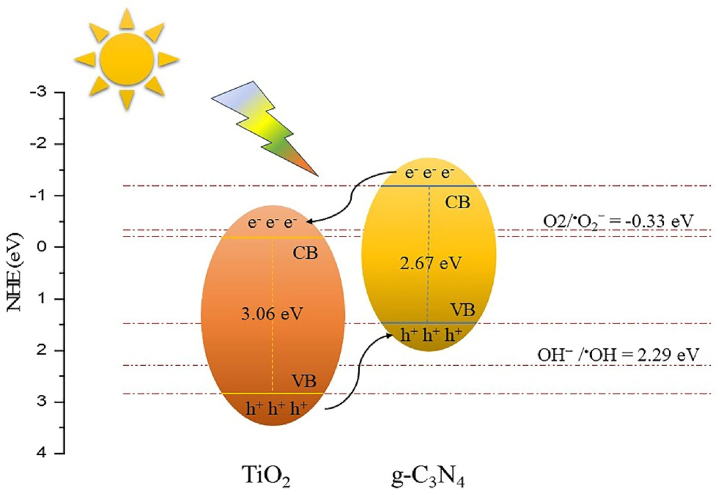
Schematic of the band structure and charge transfer mechanism of TCN type II.

As Fig. 9 illustrates, the degradation efficiency and concentration of methylene blue (MB) showed changes after introducing scavengers. After 1 h of visible light irradiation, the degradation efficiency decreased from 94.58 % to 94.15 %, 41.73 %, and 39.61 %, respectively. According to the results, the addition of IPA had no significant impact on the degradation performance, suggesting that hydroxyl radicals (O· H) have a negligible role in the photocatalytic degradation of MB. Whereas superoxide radicals (O·2‐) and holes ($h^{+}$) play a dominant role in the dye degradation of methylene blue in the presence of TCN-CA/U nanocomposite under visible light. This scavenger study provides valuable insights into the specific radicals involved in the photocatalytic process, enhancing our understanding of the mechanism behind the improved degradation of organic pollutants. After loading the TiO_2_ nanocomposite onto the surface of g-C_3_N_4_, it provides more hydrogen evolution active sites and speeds electron transformation, increasing the catalyst's activity.

To further comprehension of the photocatalytic mechanism, Mulliken's electronegativity theory can be used to calculate the conduction band (CB) and valence band (VB) levels in the heterojunction of TCN. Equations can be used to predict the potential of g-C_3_N_4_ VB and TiO_2_ edges [64].

(10) $$E_{\text{VB}}=X+\left(E_{g}-E^{e}\right)$$

(11) $$E_{\text{CB}}=E_{\text{VB}}-E_{g}$$

Where X is the semiconductor's absolute electronegativity, E_CB_ and E_VB_ are its conduction and valence band edge potentials, Eg is its band gap, and E^e^ is its energy of free electrons on the hydrogen scale. G-C_3_N_4_ and TiO_2_ have electronegativity values of 4.64 eV and 5.81 eV, respectively [27]. These computations yielded the following results: E_VB_ and E_CB_ of g-C_3_N_4_ were calculated to be 1.475 eV and −1.195 eV, and that of TiO_2_ were found to be 2.84 eV and −0.22 eV, respectively.

**Fig. 10:**
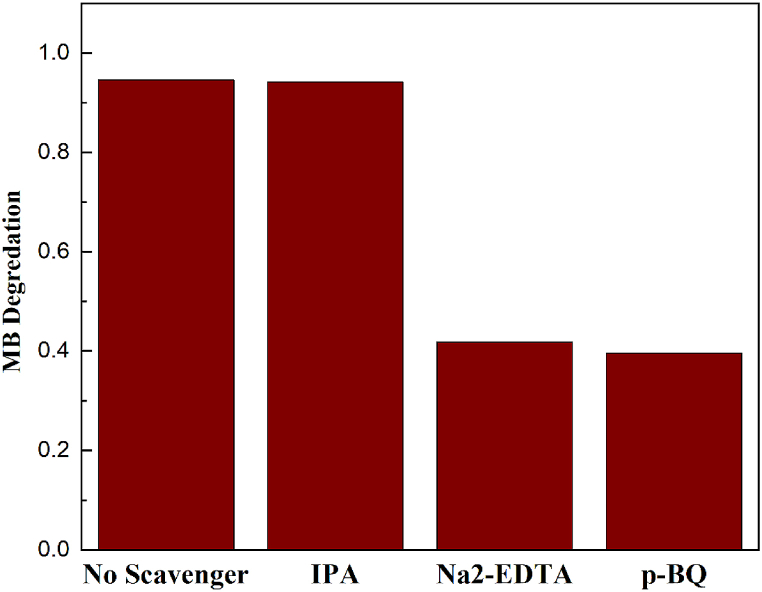
Photodegradation of the MB dye by the TCN-CA/U nanocomposite in the presence of a series of scavengers.

The probable mechanism of enhanced photocatalytic performance of pollutants decontamination of the TCN nanocomposites is depicted in Fig. 10. According to the band gap of synthesized pristine materials, electron-hole pairs were excited only in the g-C_3_N_4_ phase under visible light irradiation (Eq. (12)). In contrast, TiO_2_ does not have the capability of generating electron-hole pairs under the same condition due to its wide band gap (>3.0 eV). For effectively generating (O·2‐), the band edge of the semiconductor must be more negative than the redox potential $O_{2}$/ $O_{2}^{‐}$ (0.33 eV vs. NHE). Due to the position of the conduction bands, the CB edge of TiO_2_ is lower than the oxidation potential of (O_2_/O·2‐) and consequently, superoxide radicals (O·2‐) cannot be generated on the surface of TiO_2_ [65,66]. However, based on the CB edge of g-C_3_N_4_, some of the electrons are captured by oxygen adsorbed on the surface and producing superoxide radicals (O·2‐) with strong oxidative power (Eq. (13)) [54]. Furthermore, the conduction band edge of g-C_3_N_4_ (−1.195 eV vs. NHE) is more negative than that of TiO_2_ (−0.22 eV vs. NHE), then the photogenerated electrons in CB of g-C_3_N_4_ rapidly transferred to the CB of TiO_2_, which led to enhanced carrier separation within the g-C_3_N_4_ phase. In parallel, the photogenerated holes in the valence bands of the g-C_3_N_4_ also contribute to oxidization. On the other hand, the standard redox potential for the OH⁻/ O· H is 2.29 eV versus NHE, so the valence band position must be more positive to allow h⁺ to generate OH radicals. Wherein the transport directions of photogenerated holes should be opposite, moving from TiO_2_ to the surface of g-C_3_N_4_, and these photogenerated holes in the TiO_2_ valence band potentially have a high reduction potential, which can reduce OH^−^ to strong oxidizing hydroxyl radicals (OH·), and then react with the pollutants that adsorbed on the catalyst surface, as shown in (Eq.s (14), (15))). However, the VB of the TiO_2_ does not form holes under visible light and subsequently, $\left(h_{\text{VB}}^{+}\right)$ on the VB cannot oxidize H_2_O/O $H^{‐}$ to O· H [64]. Herein, the formed Type-II heterojunction between g-C_3_N_4_ and TiO_2_ enhances the photocatalytic activity of TCN nanocomposites for the degradation of dye pollutants where O·2‐ and $h_{\text{VB}}^{+}$ are the primary reason for the decontamination of dye pollutants under visible light (Eq. (16)) [66,67]. This proposed mechanism is in agreement with the results from photocatalytic scavenger experiments.

The key photocatalytic reactions can be described as follows [68]:

(12) $$photocatalyst+h\lambda \;\to \;e_{\text{CB}}^{‐}+h_{\text{VB}}^{+}$$

(13) $$O_{2\;}+e_{\text{CB}}^{‐}\;\to \;O_{2}^{‐}$$

(14) $$O_{2}^{‐}+H_{2}\;O\;\to \;H\;O_{2}^{\cdot }+O\;H^{‐}$$

(15) $$O\;H^{‐}+h_{\text{VB}}^{+}\to \;O\;H$$

(16) $$O_{2}^{‐}+h_{\text{VB}}^{+}+dye\;pollutants\;\to \;Degradation\;products\;\left(C\;O_{2}+H_{2}\;O\right)$$

This mechanism explains how the TCN-CA/U heterojunction effectively harnesses visible light for photocatalytic degradation, resulting in the removal of organic pollutants and the generation of benign degradation products.

## 8 Conclusion

In summary, the TCN nanocomposites were successfully synthesized via a solution combustion method. Various fuels, including citric acid, glycine, urea, and mixtures of citric acid with urea or glycine, were employed to investigate impacting each on the photocatalytic performance of the nanocomposites. Among fuels, citric acid showed higher photocatalytic performance due to its chelation characteristics and lower decomposition temperature, which produced larger TiO2 particles on the g-C₃N₄ surface. Furthermore, the fuel mixture equipped nanocomposites with higher porosity and more efficient charge transfer, which are beneficial for photocatalytic activity. Among the synthesized nanocomposites, TCN-CA/U exhibited the highest photocatalytic performance, achieving a remarkable 97.03 % degradation rate of methylene blue (MB) within 120 min of visible light irradiation. Comparatively, this performance was 13.5 times better than TiO_2_ and 3 times more effective than g-C_3_N_4_. The enhanced photocatalytic performance of TCN nanocomposites is attributed to the formation of a type II heterojunction that reduced electron-hole recombination, a narrowed band gap due to synergetic combustion reaction and introduced carbon dopants to the structure of nanocomposites, and the higher specific surface area because of the releasing a significant amount of gas byproducts during synthesized route. This study demonstrated that synthesized TCN nanocomposites via the solution combustion method are promising materials for the photocatalytic degradation of organic pollutants and present an efficient approach for wastewater treatment under visible light irradiation.

## CRediT authorship contribution statement

**S. Delafrouz:** Writing – review & editing, Writing – original draft, Investigation, Formal analysis, Data curation. **M. Hasheminiasari:** Writing – review & editing, Validation, Supervision, Methodology, Conceptualization. **S. Alamolhoda:** Validation, Supervision, Methodology, Conceptualization. **S.M. Masoudpanah:** Validation, Supervision, Methodology.

## Declaration of Competing Interest

The authors declare that they have no known competing financial interests or personal relationships that could have appeared to influence the work reported in this paper.
